# GWAS reveal novel IgA nephropathy risk loci

**DOI:** 10.18632/oncotarget.4632

**Published:** 2015-06-24

**Authors:** Jia Nee Foo, Jianjun Liu, Xue-Qing Yu

**Affiliations:** Human Genetics, Genome Institute of Singapore, Singapore, Singapore

**Keywords:** Chromosome Section, IgA nephropathy, GWAS, genetic risk loci and biological mechanism

IgA nephropathy, also known as Berger's disease, is the most common primary glomerulonephritis and a major cause of end stage renal disease (ESRD). IgA nephropathy typically presents as macroscopic haematuria (blood in the urine), often following a mucosal infection, and diagnosis is made based on a renal biopsy which shows deposition of IgA-containing immune complexes in the mesangial area of glomeruli and histopathological lesions such as mesangial cell proliferation and accumulation of extracellular matrix. It leads to progressive loss of kidney function and between 20 to 40% of cases will progress to ESRD within 20 years of disease onset [1]. There is substantial variation in prevalence of IgA nephropathy globally, with highest frequency in Asian populations, modest frequency in the European population and lowest frequency in the African population. These differences, together with evidence of familial clustering and renal abnormalities among relatives of cases, strongly suggest the presence of a substantial genetic contribution to disease development [1].

Genome-wide association studies (GWAS) have identified common variants within several loci (chromosomal regions) associated with IgA nephropathy risk. We conducted the most recent study, analyzing a total of 8,313 cases and 19,680 controls from the Han Chinese population across four stages [2]. This is the largest sample collection to date. Separately, a recent study by Kiryluk et al. analysed 7,658 cases and 12,954 controls, out of which 3,685 cases and 2,682 controls are East Asians and the rest are Europeans [3]. These two studies identified six novel loci over the previously identified five (namely 1q32 containing *CFHR3*-*CFHR1* genes, 6p21 containing human leucocyte antigen class II genes, 8p23 containing the defensin gene cluster, 17p13.1 containing *TNFSF13* and 22q12 containing *HORMAD2*), bringing the total to eleven [4,5].

We discovered novel associations at *ST6GAL1* on 3q27.3, *ACCS* on 11p11.2 and *ODF1- KLF10* on 8q22.3 [2], validated the recently reported association at *ITGAX- ITGAM* (16p11.2) and moderately replicate the reported associations at *VAV3* (1p13) and *CARD9* (9q34) [3]. Most of these loci, including two of our newly discovered ones *ST6GAL1* and *UBR5* (near *ODF1- KLF10)* implicate genes involved in innate immunity and IgA production, in particular mucosal immunity in the gut [2,3]. Several loci are shared with a variety of other autoimmune diseases [2,3]. The third locus harbouring the genes *ACCS* and *EXT2* may implicate a new pathway involved in cell proliferation and heparan sulfate biosynthesis, which may influence the development of lesions in the kidneys, but further studies will be needed to confirm this.

Out of the eleven loci, three contain common nonsynonymous variants that could potentially explain the association signals (*ITGAX* p.Pro517Arg*, CARD9* p.Ser12Ile and *TNFSF13* p.Asn96Ser) [2-5]. However, these variants, as well as those at several other loci (including *ST6GAL1and ACCS*), are strongly associated with mRNA expression levels of the respective nearby genes in peripheral blood cells [6]. Many of the risk alleles tag SNPs that lie in putative regulatory regions identified by the Encyclopaedia of DNA Elements (ENCODE) project [7]. This suggests that many of these common risk alleles are influencing disease risk through their effects on the regulation of gene expression.

In addition, variants at several loci including *ST6GAL1, ACCS*, and *ITGAX* showed a trend of increasing risk allele frequencies from the African, European to Asian samples of the HapMap project, suggesting that they may contribute cumulatively to geographical differences in the genetic susceptibility and thus the disease prevalence of IgA nephropathy [2,3]. As observed by Kiryluk et al., these differences also correlate with documented geographical variation in helminth diversity, which includes intestinal parasites, thus suggesting that there might have been positive selection for the IgA nephropathy risk alleles in Asia to cope with the increased regional diversity of these pathogens [3].

In the largest study on IgA nephropathy to date, we have discovered three new loci, validated the recently reported ones and provided additional support for the previously-identified defensin gene cluster locus by identifying two novel independent associations within the locus. We estimate that these novel association signals explain about 1.7% of the disease variance and 5.5% of the variance in combination with the previously published loci [2-5]. Genes within these loci have provided important insight into the potential biological mechanisms and pathways that influence genetic risk to IgA nephropathy.

Moving forward, we need to continue mining the genome for new loci, by increasing sample size and therefore statistical power to detect variants with smaller effects, and to evaluate the roles of low frequency and rare variants by deep resequencing. More importantly, we need to identify the genetic factors that influence intermediate clinical sub-phenotypes and the risk of progression to ESRD. This will require the integration of the genomic profile with other omics profiles (e.g. transcriptomics, metabolomics and immunomics) in patients with long-term clinical follow-up to better understand the factors underlying inter-individual variability, not only in disease susceptibility, but also in the long-term prognosis and healthcare requirements [1].
